# Potential impact of a nine-valent vaccine in human papillomavirus related cervical disease

**DOI:** 10.1186/1750-9378-7-38

**Published:** 2012-12-29

**Authors:** Beatriz Serrano, Laia Alemany, Sara Tous, Laia Bruni, Gary M Clifford, Thomas Weiss, Francesc Xavier Bosch, Silvia de Sanjosé

**Affiliations:** 1 Unit of Infections and Cancer (UNIC), Cancer Epidemiology Research Program (CERP), IDIBELL, Institut Català d’Oncologia (ICO) - Catalan Institute of Oncology, Gran Via de l’Hospitalet, 199-203, L’Hospitalet de Llobregat,, Barcelona, Spain; 2 CIBER Epidemiología y Salud Pública, CIBERESP,, Pamplona, Spain; 3 International Agency for Research on Cancer, Lyon, France; 4 Global Health Outcomes, Merck & Co., Inc., West Point, PA, , Barcelona, Spain

**Keywords:** Human papillomavirus, Cervical cancer, Genotype, Epidemiology, Human papillomavirus vaccines

## Abstract

**Background:**

Information on human papillomavirus (HPV) type distribution is necessary to evaluate the potential impact of current and future HPV vaccines. We estimated the relative contribution (RC) to invasive cervical cancer (ICC) and precancerous cervical lesions of the nine HPV types (HPV 6/11/16/18/31/33/45/52/58) included in an HPV vaccine currently under development.

**Methods:**

Estimations on ICC were based on an international study of 8,977 HPV positive cases and estimations on precancerous cervical lesions were extracted from a published meta-analysis including 115,789 HPV positive women. Globocan 2008 and 2010 World Population Prospects were used to estimate current and future projections of new ICC cases.

**Results:**

RC of the 9 HPV types in ICC was 89.4%, with 18.5% of cases positive for HPV 31/33/45/52/58. Regional variations were observed. RCs varied by histology, ranging between 89.1% in squamous cell carcinomas (SCC) and 95.5% in adenocarcinomas (ADC). HPV 16/18/45 were detected in 94.2% of ADC. RC of the 9 types altogether decreased with age (trend test p < 0.0001), driven by the decrease in older ages of HPV 16/18/45. In contrast, the RC of HPV 31/33/52/58 increased with age. Due to population growth alone, projected estimates of ICC cases attributable to the 9 types are expected to rise from 493,770 new cases in 2012 to 560,887 new cases in 2025.

The RCs of individual high risk HPV types varied by cytological and histological grades of HPV-positive precancerous cervical lesions, and there was an under representation of HPV 18 and 45 compared to ICC.

**Conclusions:**

The addition of HPV 31/33/45/52/58 to HPV types included in current vaccines could prevent almost 90% of ICC cases worldwide. If the nine-valent vaccine achieves the same degree of efficacy than previous vaccines, world incidence rates could be substantially reduced.

## Background

Cervical cancer and other HPV related cancers represent an important global public health problem, both in more and less developed countries. Cervical cancer is the third most common cancer among women worldwide, with an estimated 530,000 new cases and 275,000 new deaths in 2008. More than 85% of the global cervical cancer burden occurs in developing countries and in 12 of the 22 regions of the world, cervical cancer remains the first or second most common female cancer [1].

HPV carcinogenicity has been convincingly established for cervical cancer and it is generally accepted that HPV infection is necessary for the development of invasive cervical cancer (ICC) [2]. The International Agency for Research on Cancer (IARC) has classified HPV 16 and 18 as cervical carcinogens since 1995. By 2011 the group was expanded to include HPV 31/33/35/39/45/51/52/56/58/59 [3]. It is estimated that over 50-80% of sexually active women will be infected with one or more genital HPV types during their lives, with peak prevalence in young sexually active individuals [3-5].

HPV prophylactic vaccines using virus like particles (VLP) have been recognized as a major advance and the most effective intervention to control for HPV and cervical cancer [4]. Current licensed HPV vaccines, a bivalent (CervarixTM) and a quadrivalent HPV vaccine (Gardasil®) are designed to prevent HPV infection and HPV-related disease. CervarixTM was designed to prevent infection by HPV types 16/18, which cause about 70 percent of cervical cancer cases [6,7], and Gardasil® targets the same two cancer causing types and, in addition, is intended to prevent infection by HPV 6/11, which cause close to 90% of external genital wart cases [8]. These vaccines are near 100% effective in stimulating the immune system against the strains targeted in the vaccine when administered in a 3 dose course [9]. Given the high safety and efficacy of both vaccines, many countries have licensed and some have already included them in their national immunization programs, but they are yet to reach women in most low-income countries [10]. In addition, the GAVI Alliance has announced country applications for national introduction of HPV at very low price aiming feasibility and sustainability of such vaccines even in the very poor settings [11,12].

Future impact of HPV vaccination with current commercialized vaccines has been largely modelled [13,14]. Models predict a substantial reduction in prevalence of specific HPV 16/18 infections, followed by a reduction in cervical abnormalities and a final reduction in ICC, if coverage is high (>70%) and vaccine induced protection lasts for at least 10 years [4]. The greatest impact is expected in low-income and middle-income countries where there is no screening or only limited screening for cervical cancer and where the highest burden of cervical disease is generally observed [4]. Impact of vaccination with current HPV vaccines in low resource settings, without any other preventive actions, is estimated to potentially reduce cancer risk by 40–50% at 70% vaccination coverage, with a potential reduction in the next decade of more than 4 million deaths among vaccinated women [14,15].

After HPV 16/18, data confirm HPV 31/33/35/45/52/58 as the most frequently detected types in ICC worldwide [6,7]. Merck is developing a recombinant nine-valent vaccine, code named V503, that also uses virus like particles (VLP). V503 contains 5 additional HPV types (31/33/45/52/58) to the 4 HPV types previously included in Gardasil® and has successfully completed several studies. Phase III studies are ongoing [16].

The aim of the present article is to estimate the potential impact of a new nine-valent vaccine in HPV-related cervical disease by summarizing the evidence on specific relative contribution (RC) of the 9 HPV types (HPV 6/11/16/18/31/33/45/52/58) in ICC and precancerous cervical lesions, in the pre-vaccination era.

## Methods

To estimate the RC of the 9 HPV types included in the V503 Merck's investigational HPV vaccine, we used data from an international study on HPV in ICC [7] and the most recent meta-analysis in precancerous cervical lesions available in the literature [17].

### Cervical cancer

The estimations on RC in ICC were based on data from a retrospective cross-sectional study carried out at Catalan Institute of Oncology, Spain (ICO) in collaboration with DDL laboratory, The Netherlands, to estimate the distribution of HPV types in women with ICC between 1949 and 2009 [7]. Briefly, the study included information from 10,575 ICC cases, and 8,977 (85%) of them were positive for HPV DNA. Paraffin-embedded specimens from consecutive cases (aged 16–97 years) with ICC were obtained from hospital pathology archives in 38 countries—Europe (Bosnia-Herzegovina, Croatia, Czech Republic, France, Greece, Italy, Netherlands, Poland, Portugal, and Spain); North America (USA); Central and South America (Argentina, Brazil, Chile, Colombia, Guatemala, Honduras, Mexico, Paraguay, Peru, and Venezuela); Africa (Algeria, Mozambique, Nigeria, and Uganda); Asia (Bangladesh, China, India, Israel, Japan, South Korea, Kuwait, Lebanon, Philippines, Taiwan, Thailand, and Turkey); and Oceania (Australia). After the blocks were processed, inclusion criteria were a pathological confirmation of a primary ICC of epithelial origin in the tissue sample selected for analysis of HPV DNA. HPV DNA detection was done by PCR with SPF-10 broad-spectrum primers followed by DNA enzyme immunoassay and genotyping with a reverse hybridization line probe assay - LiPA25 that detects 25 high-risk (HR) and low-risk (LR) HPV types (6/11/16/18/31/33/34/35/39/40/42/43/44/45/51/52/53/54/56/58/59/66/68/70/74). Sequence analysis was done to characterize HPV-positive samples with unknown HPV types. If no HPV type could be attributed after DNA sequencing, the HPV was labelled HPV undetermined (52 cases out of 8,977 (0.6 %)). All protocols were approved by the local and ICO ethics committees, and the entire study progress was overseen by an international steering committee.

RC of the 9 HPV types included in the nine-valent vaccine (HPV 6/11/16/18/31/33/45/52/58) in ICC was expressed as the proportion of women positive for a given type among all the HPV-positive samples. The availability of HPV-disaggregated data made possible the calculation of RCs for HPV types alone or for the groups of HPV types. Multiple infections were added to single types in accordance with a proportional weighting attribution [18,19]. For example, if two ICC lesions found to test positive for both HPV 16 and 45 in a region of the world, and there were 9 cases infected by HPV-16 as a single type and 1 case infected by HPV 45 as a single type, then [2*9/(9 + 1)] or 1.8 of these two multi-type infected lesions would be attributed to HPV 16 and [2*1/(9 + 1)] or 0.2 attributed to HPV 45.

Subjects were classified into 10 geographical regions defined by the United Nations Population Divisions (Africa, Asia, Eastern Asia, Western/Central Asia, Europe, North America, Latin America, Central America, South America and Oceania). RC was provided for the following histological categories: Squamous Cell Carcinoma (SCC), Adenocarcinoma (ADC), Adenosquamous Cell Carcinoma (ADSCC) and Other histology that included undifferentiated, neuroendocrine, not otherwise specified, basal adenoid and cystic adenoid carcinomas. Estimations of HPV RCs by age groups were also provided. Evaluations of the trends by age were determined by trend test analysis. Statistically significant p-value for this analysis was set at 0.05 level.

Projections of new ICC cases attributed to HPV 16/18 and HPV 31/33/45/52/58 were calculated for year 2012 and projected to year 2025, worldwide and by region. Estimations were based on the following assumptions and data sources: a) Oncogenic HPV types may be detected in virtually all cases of cervical cancer, being generally accepted that the virus is necessary for the development of cancer, so the RC of all HPV types altogether is assumed to be 100%. Therefore, we assumed that the attributional fraction (AF) for each HPV type in ICC corresponded to the RC in ICC. The overall RC was used as the world AF, without weighting regional estimations; b) Globocan 2008 incidence rates were used with the assumption that these rates will apply in the future [1]; c) We used the appropriate population forecast available in the latest World Population Prospects (revision 2010) [20]. Impact of vaccination was not taken into account.

### Precancerous cervical lesions

The estimations on RCs in precancerous cervical lesions were based on data from a recent IARC meta-analysis [17]. Briefly, this meta-analysis included information on HPV type distribution in 115,789 HPV-positive women from 423 PCR-based studies worldwide. Inclusion criteria were use of broad-spectrum consensus PCR-assays based on the primers MY09/11, PGMY09/11, GP5+/6+, SPF10, SPF1/GP6 or L1C1/L1C2, and information of overall and type-specific HPV prevalence, by strata of cyto- and/or histo-pathological cervical diagnoses. LR HPV types were not included in the meta-analysis, so specific information for HPV 6 and 11 was not provided. Data were extracted from the manuscript for 12,983 cases (52% of which were HPV-positive) of atypical squamous cells of undetermined significance (ASCUS), 17,805 cases (76% HPV-positive) of low-grade squamous intraepithelial lesions (LSIL), and 7,743 cases (85% HPV-positive) of high-grade SIL (HSIL) diagnosed cytologically, 11,043 cases (73% HPV-positive) of cervical intraepithelial neoplasia grade 1 (CIN1), 4,754 cases (86% HPV-positive) of CIN2 and 11,618 cases (93% HPV-positive) of CIN3 diagnosed histologically. As described in the manuscript [17], in order to retain appropriate sample size for comparisons between regions, precancerous cervical lesions were collapsed in two categories; Low-grade, encompassing ASCUS, LSIL and CIN1; and High-grade, including HSIL, CIN2 and CIN3.

The RCs of the 7 individual HR HPV types (16/18/31/33/45/52/58) were expressed as the proportion of samples positive for a given type among all the HPV-positive samples tested for the mentioned type, therefore denominators vary by type. Individual-level data on HPV types in multiple infections were not available, so RC includes single and multiple infections and the total sum of RCs of the 7 HR HPV can exceed 100%. Subjects were classified into seven geographical regions defined by the United Nations Population Divisions (Africa, Eastern Asia, Western/Central Asia, Europe, North America, South/Central America and Oceania) [17].

In an attempt to estimate a combined RC estimation of the 7 HR HPV types altogether for CIN2/3 or HSIL lesions similar to that in ICC, we used Smith et al. meta-analysis [21] (for studies published from 2002 to 2006) and Guan et al. meta-analysis [17] (for studies from 2006–2011) as data sources to identity papers providing detailed data for single and multiple infections. We established the following criteria for eligibility: (i) inclusion of at least 75 cases of HSIL, CIN2 or CIN3, (ii) use of broad-spectrum PCR, and (iii) reporting of type-specific HPV prevalence for at least the 7 HR HPV genotypes (HPV 16/18/31/33/45/52/58) included in the nine-valent Merck’s vaccine. Forty seven studies met inclusion criteria, including a total of 14,039 cases with an overall HPV prevalence of 89.7%. Assessment of the RC of the 7 HR HPV types altogether was only possible in 13 studies (including 4,101 cases) where individual-level data on HPV types in multiple infections was available.

## Results

### Cervical cancer

Combined RC of the 9 HPV types included in the nine-valent vaccine (HPV 16/18/31/33/45/52/58/6/11) worldwide was 89.4% (95%CI: 88.8-90.1), with some regional variations, from 84.6% (95%CI: 81.9-87.1) in Central America to 95.5% (95%CI: 91.2-98.2) in North America. HPV 16/18 were the most common types accounting for almost 70% worldwide, except for Central America and North America where a RC of 64.4% (95%CI: 60.9-67.8) and 78.8% (95%CI: 71.6-84.8) were observed respectively. The additional RC of the other 5 high risk HPV types (HPV 31/33/45/52/58) was 18.5% (95%CI: 17.7-19.3); ranging from 8.8% (95%CI: 5.0-14.1) in Oceania to 22.3% (95%CI: 20.4-24.3) in Eastern Asia. HPV 45 was the most frequently detected type after HPV 16 and 18 worldwide except in Eastern Asia where RCs of HPV 52/58 were higher, and in Europe where RC of HPV 33 was higher. RC of HPV 45 was especially prominent in Africa (9.9%; 95%CI: 7.6-12.8), Western Asia (7.0%; 95%CI: 5.4-9.0) and Latin-America (6.8%; 95%CI: 5.9-7.7). HPV 31 was relatively more prevalent in Latin-America (4.9%; 95%CI: 4.2-5.7) and HPV 33 was more prevalent in Europe (5.7%; 95%CI: 4.7-6.8), as previously remarked. Infections by HPV 52/58 were more usually diagnosed in Eastern Asia (5.3%; 95%CI: 4.3-6.4 and 5.0%; 95%CI: 4.1-6.2, respectively). Addition of HPV 6/11 did not modify the cancer burden estimates (RC: 0.1%) (Table 1).

**Table 1:** Relative contribution of HPV 16/18/31/33/45/52/58 in cases of ICC HPV-positive, by region

	**World**	**Africa**	**Asia**	**Western/ Central Asia**	**Eastern Asia**	**Latin America**	**South America**	**Central America**	**North America**	**Europe**	**Oceania**
	**(n = 8,977)**	**(n = 544)**	**(n = 2,641)**	**(n = 836)**	**(n = 1,805)**	**(n = 3,404)**	**(n = 2,629)**	**(n = 775)**	**(n = 160)**	**(n = 2,058)**	**(n = 170)**
	**RC % (95%CI)**	**RC % (95%CI)**	**RC % (95%CI)**	**RC % (95%CI)**	**RC % (95%CI)**	**RC % (95%CI)**	**RC % (95%CI)**	**RC % (95%CI)**	**RC % (95%CI)**	**RC % (95%CI)**	**RC % (95%CI)**
***Combinations of HPV types***											
9 HPV types*	89.4 (88.8-90.1)	86.9 (83.8-89.7)	91.5 (90.4-92.5)	92.0 (89.9-93.7)	91.3 (89.9-92.6)	88.2 (87.0-89.2)	89.2 (87.9-90.3)	84.6 (81.9-87.1)	95.5 (91.2-98.2)	89.1 (87.7-90.4)	89.4 (83.8-93.6)
**16/18**	70.8 (69.8-71.7)	70.3 (66.2-74.0)	71.6 (69.8-73.4)	77.5 (74.5-80.3)	68.9 (66.7-71.1)	68.2 (66.6-69.8)	69.4 (67.6-71.1)	64.4 (60.9-67.8)	78.8 (71.6-84.8)	72.8 (70.8-74.7)	78.2 (71.3-84.2)
**31/33/45/52/58**	18.5 (17.7-19.3)	16.7 (13.7-20.1)	19.8 (18.3-21.3)	14.5 (12.2-17.0)	22.3 (20.4-24.3)	19.8 (18.5-21.2)	19.6 (18.1-21.2)	20.3 (17.5-23.3)	16.9 (11.4-23.6)	16.2 (14.6-17.8)	8.8 (5.0-14.1)
**Other Types**	10.6 (9.9-11.2)	13.0 (10.3-16.2)	8.5 (7.5-9.6)	8.0 (6.3-10.1)	8.7 (7.4-10.1)	11.8 (10.8-13.0)	10.9 (9.7-12.1)	15.4 (12.9-18.1)	4.6 (1.8-8.8)	10.9 (9.6-12.3)	10.6 (6.4-16.2)
***Specific HPV types***											
**16**	60.5 (59.5-61.6)	47.7 (43.3-51.9)	60.5 (58.6-62.3)	66.7 (63.3-69.8)	57.6 (55.3-59.9)	59.2 (57.5-70.9)	61.3 (59.4-63.2)	52.0 (48.4-55.6)	71.9 (64.2-78.9)	65.5 (63.4-67.6)	58.6 (51.0-66.3)
**18**	10.3 (9.6-10.9)	22.6 (19.2-26.4)	11.2 (10.0-12.4)	10.8 (8.9-13.2)	11.3 (9.9-12.9)	9.1 (8.1-10.1)	8.1 (7.1-9.2)	12.5 (10.3-15.1)	6.9 (3.48-11.8)	7.3 (6.2-8.5)	19.9 (14.3-26.8)
**31**	3.7 (3.3-4.1)	1.9 (0.9-3.4)	3.0 (2.4-3.8)	2.8 (1.6-4.0)	3.2 (2.4-4.1)	4.9 (4.2-5.7)	6.5 (5.6-7.6)	4.3 (3.0-5.9)	3.4 (1.0-7.1)	3.4 (2.7-4.3)	0.7 (0.0-3.2)
**33**	3.8 (3.4-4.3)	1.5 (0.6-2.9)	3.5 (2.8-4.3)	2.5 (1.6-3.8)	4.0 (3.1-4.9)	3.5 (3.2-4.5)	5.0 (4.3-6.0)	2.9 (1.9-4.4)	3.1 (1.0-7.1)	5.7 (4.7-6.8)	2.1 (0.4-5.1)
**45**	5.9 (5.4-6.4)	9.9 (7.6-12.8)	5.5 (4.7-6.5)	7.0 (5.4-9.0)	4.9 (3.9-6.0)	6.8 (5.9-7.7)	3.7 (3.0-4.4)	7.5 (5.7-9.6)	5.4 (2.6-10.4)	3.9 (3.1-4.8)	5.5 (2.5-9.8)
**52**	2.8 (2.5-3.2)	2.6 (1.4-4.3)	3.8 (3.1-4.6)	0.8 (0.3-1.7)	5.3 (4.3-6.4)	2.7 (2.2-3.3)	3.0 (2.4-3.7)	1.6 (0.9-2.9)	2.9 (1.0-7.1)	1.9 (1.4-2.6)	0.6 (0.0-3.2)
**58**	2.3 (2.0-2.6)	0.7 (0.2-1.9)	3.9 (3.2-4.7)	1.3 (0.7-2.3)	5.0 (4.1-6.2)	2.0 (3.3-2.5)	1.4 (1.0-2.0)	3.9 (2.6-5.5)	1.9 (0.39-5.4)	1.3 (0.9-1.9)	0.0 (0.0-2.2)
**6**	0.1 (0.1-0.2)	0.0 (0.0-0.7)	0.0 (0.0-0.2)	0.0 (0.0-0.4)	0.1 (0.0-0.3)	0.1 (0.0-0.3)	0.1 (0.0-0.3)	0.0 (0.0-0.5)	0.0 (0.0-2.3)	0.2 (0.0-0.4)	1.8 (0.4-5.1)
**11**	0.0 (0.0-0.1)	0.0 (0.0-0.7)	0.0 (0.0-0.2)	0.0 (0.0-0.4)	0.1 (0.0-0.3)	0.0 (0.0-0.2)	0.0 (0.0-0.2)	0.0 (0.0-0.5)	0.0 (0.0-2.3)	0.0 (0.0-0.2)	0.0 (0.0-2.2)

"ICC": Invasive cervical cancer; “RC”: Relative Contribution; “95%CI”: 95% Confidence Interval; * ”9 HPV types” includes the ones in nine-valent vaccine: HPV 16/18/31/33/45/52/58/6/11.

Additional information: Available data are from unvaccinated women (pre-vaccination period).

Type specific RC estimations: Numerator = single infections + proportional attribution of multiple types; Denominator = HPV DNA positive cervical cancer cases.

**Figure 1:**
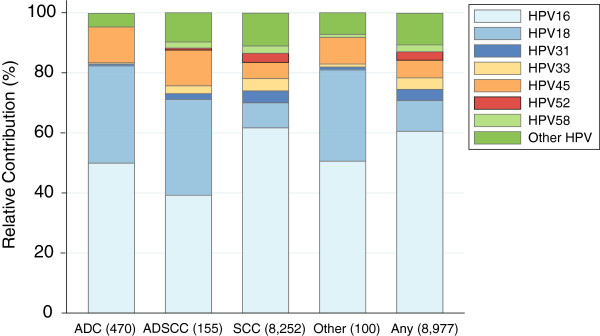
**Relative contribution of HPV 16/18/31/33/45/52/58 in cases of ICC HPV-positive, by histology.** "ICC": Invasive cervical cancer; “SCC”: Squamous cell carcinoma; “ADC”: Adenocarcinoma; “ADSCC”: Adenosquamous cell carcinoma; “Other”: Includes undifferentiated, neuroendocrine, not otherwise specified, basal adenoid and cystic adenoid carcinomas. Additional information: Available data are from unvaccinated women (pre-vaccination period). Type specific relative contribution estimations: Numerator = single infections + proportional attribution of multiple types; Denominator = HPV DNA positive cervical cancer cases. The number of cases by histology is shown in “( )”. Specific information on HPV 6 and 11 is not included due to the low relative contribution of these types.

Regarding histological diagnosis, the combined RC of the 9 HPV types ranged from 89.1% (95%CI: 88.4-89.7) in SCC to 95.5% (95%CI: 93.3-97.2) in ADC. HPV 16/18/45 were consistently the three most common types in all histological categories accounting for 94.2% (95%CI: 91.7-96.2) of ADC cases compared to the 75.4% (95%CI: 74.5-76.3) contribution in SCC. A greater contribution of HPV 18/45 was observed in ADC, ADSCC and other histologies compared to that in SCC and conversely, HPV 16 RC was higher in SCC. Substantial differences in the RCs by histological category were observed for the other HPV types, with much higher contributions of HPV 31/33/52/58 in SCC (RC: 13.6%) and ADSCC (RC: 7.2%) compared to the RC of less than 1% and 3% observed in ADC cases and other histologies, respectively (Figure 1).

**Figure 2:**
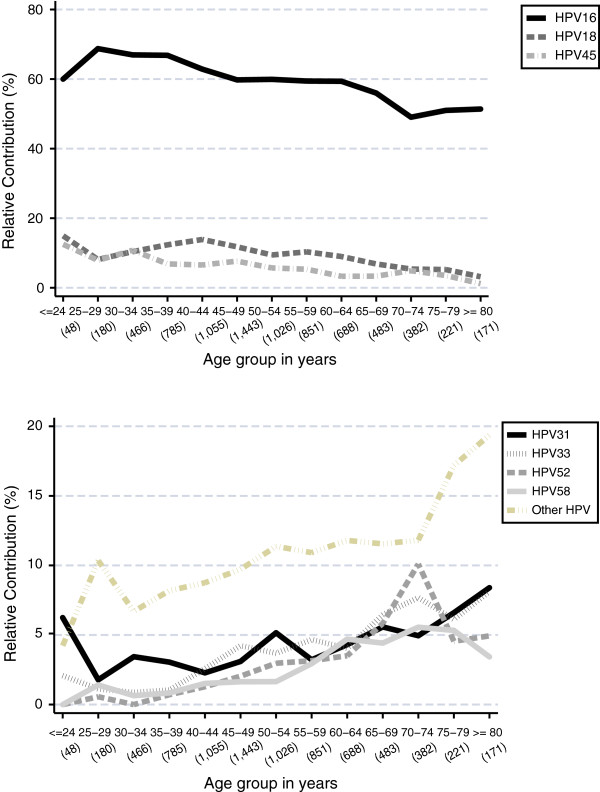
**Relative contribution of HPV 16/18/45/31/33/52/58 and other types in ICC cases HPV-positive, by age group.** "ICC": Invasive cervical cancer; Additional information: Available data are from unvaccinated women (pre-vaccination period). Type specific relative contribution estimations: Numerator = single infections + proportional attribution of multiple types; Denominator = HPV DNA positive cervical cancer cases. The number of cases by age group is shown in “( )”. Specific information on HPV 6 and 11 is not included due to the low relative contribution of these types.

The RC of the 9 types altogether decreased with age (trend test p < 0.0001); however, this was mainly explained by the decrease in older ages of HPV 16/18/45. In contrast, ICC cases in women positive for HPV 31/33/52/58 increased with age (trend test p < 0.0001), although HPV 16/18/45 were also the most common types in older ages. Remarkable differences were found in ICC cases positive for HPV types other than those included in the nine-valent vaccine, with RCs ranging from 4.2% (95%CI: 0.5-14.3) in women aged less than 25 years to 19.4% (95%CI: 13.7-26.0) in women older than 80 years (Figure 2).

Without changes in prevention and control, and only due to population growth, projections for the next years reveal a growing burden of cervical cancer. Projected global estimates of ICC cases, not taking into account the impact of vaccination and assuming that all cases are related to HPV infection, are expected to rise to 627,354 new cases in 2025, with 444,167 cases attributable to HPV 16/18 and 116,061 cases attributable to HPV 31/33/45/52/58. Over 60% of cases will come from Asia, 16% from Africa and 13% from Latin America (Table 2).

**Table 2:** Burden of ICC attributable to HPV 16/18 and HPV 31/33/45/52/58 in 2012, projected to 2025; by region

**Region**	**HPV RC (%)**		**Attributable new cases (N)**			
	**16/18**	**31/33/45/ 52/58**	**Year 2012**		**Year 2025**	
			**16/18**	**31/33/45/ 52/58**	**16/18**	**31/33/45/52/58**
**World**	70.8	18.6	391,016	102,172	444,167	116,061
**Africa**	70.3	16.7	60,925	14,438	80,556	19,090
**Asia**	71.7	19.8	232,046	63,986	259,130	71,454
***Eastern Asia***	68.9	22.3	95,505	30,869	100,950	32,629
***Western/Central Asia***	77.5	14.5	145,101	27,073	171,333	31,967
**Latin-America**	68.3	19.8	48,777	14,142	54,973	15,938
***South America***	69.4	19.6	34,632	9,799	38,783	10,973
***Central America***	64.4	20.2	13,602	4,264	15,522	4,866
**North America**	78.7	16.6	9,913	2,094	10,913	2,305
**Europe**	72.8	16.2	39,880	8,867	39,924	8,877
**Oceania**	78.5	8.8	1,346	152	1,587	179

* We assume that almost all cervical cancer cases are caused by HPV infection, and thus almost 100% of cervical cancer cases are attributable to HPV infection*.*

"ICC": Invasive cervical cancer; “RC”: Relative Contribution; “N”: Number of new cases diagnosed of cervical cancer each year.

Estimated number of new ICC cases is based on GLOBOCAN 2008 estimates and World Population Prospects (2010 revision). Impact of vaccination has not been taken into account for the calculations of 2025 projections.

Specific information on HPV 6 and 11 is not included due to low relative contribution of these types.

### Precancerous cervical lesions

RCs of individual HR HPV types varied by cytological and histological grade of HPV-positive precancerous cervical lesions.

**Figure 3:**
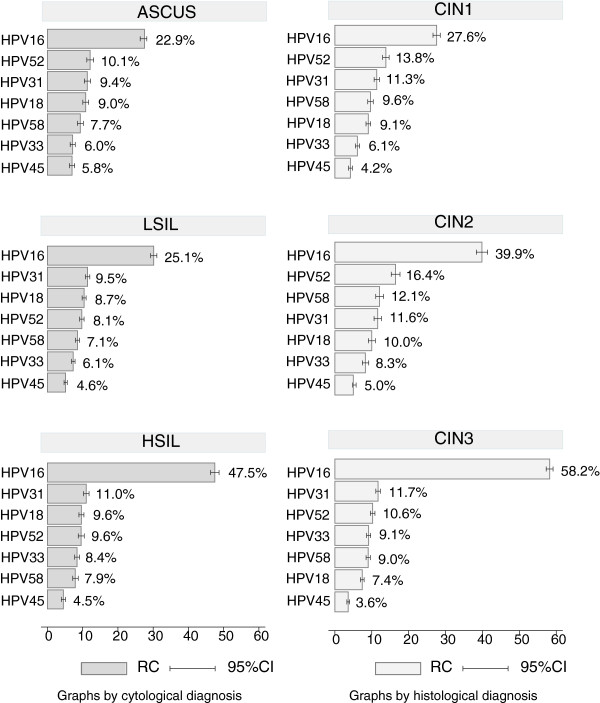
**Relative contribution of HPV 16/18/45/31/33/52/58 in precancerous cervical lesions HPV-positive, by histological and cytological diagnosis.** “CIN1”: Cervical intraepithelial neoplasia grade 1; “CIN2”: Cervical intraepithelial neoplasia grade 2; “CIN3”: Cervical intraepithelial neoplasia grade 3 (including squamous carcinoma *in situ)*; “ASCUS”: Atypical squamous cells of undetermined significance; “LSIL”: Low-grade squamous intraepithelial lesion; “HSIL”: High-grade squamous intraepithelial lesion; “RC”: Relative Contribution; “95%CI”: 95% Confidence Interval. Additional information: HPV type specific relative contribution: Numerator = includes either single or multiple infections, thus the total sum of % can exceed 100%; Denominator = ”Tested” cases that stands for the HPV/DNA positive cases from studies testing for the HPV type in question, thus denominators vary by type. The data source does not give specific information for HPV 6 and 11. 17. Data from Guan et al., IJC, 2012.

HPV 16 was the most frequently detected HR type in every grade of cervical lesion. Its importance increased with the severity of the lesion, with RCs of 47.5% (95%CI: 46.3-48.7) in HPV-positive HSIL and 58.2% (95%CI: 57.3-59.1) in HPV-positive CIN3 diagnoses. HPV 45 was consistently detected less often than other HR types included in the nine-valent vaccine in all cytological and histological categories (Figure 3).

Regional variations in the RCs of precancerous cervical lesions due to each HR HPV type were also observed. HPV 16 was the most common type in all regions in both low-grade and high-grade lesions. RC in HPV-positive high-grade lesions ranged from 30.3% to 60.3%. HPV 45 was the less frequently detected HR type in the majority of regions of the world (including Africa, where we found it to be an important HPV type in ICC). Regarding HPV-positive high-grade lesions, HPV 31 was relatively more prevalent in Western and Central Asia, and similarly to that found in ICC; HPV 52/58 showed higher RCs in Eastern Asia (Table 3).

**Table 3:** HPV 16/18/31/33/45/52/58 type-specific relative contribution in precancerous cervical lesions HPV-positive, by region

**Region**	**HPV type**	**Low grade**			**High grade**		
		**HPV-positive**	**RC**	**95%CI**	**HPV-positive**	**RC**	**95%CI**
**Africa**	**HPV-16**	517	16.8	(13.7-20.3)	251	30.3	(24.7-36.4)
	**HPV-18**	517	8.3	(6.1-11.0)	251	9.2	(5.9-13.4)
	**HPV-31**	432	6.5	(4.4-9.2)	245	8.2	(5.0-12.3)
	**HPV-33**	517	8.5	(6.3-11.3)	235	8.9	(5.6-13.3)
	**HPV-45**	432	4.4	(2.7-6.8)	245	4.1	(2.0-7.4)
	**HPV-52**	432	11.8	(8.9-15.2)	245	11.0	(7.4-15.6)
	**HPV-58**	517	10.8	(8.3-13.8)	251	11.2	(7.5-15.7)
**Eastern Asia**	**HPV-16**	2,790	21.1	(19.6-22.6)	3,693	37.9	(36.4-39.5)
	**HPV-18**	2,790	8.3	(7.3-9.4)	3,693	7.4	(6.5-8.3)
	**HPV-31**	2,552	4.4	(3.7-5.3)	3,596	6.9	(6.1-7.8)
	**HPV-33**	2,790	5.0	(4.2-5.8)	3,693	9.9	(9.0-11.0)
	**HPV-45**	2,549	1.2	(0.8-1.7)	3,148	2.0	(1.6-2.6)
	**HPV-52**	2,624	18.2	(16.8-19.8)	3,469	21.3	(20.0-22.7)
	**HPV-58**	2,781	13.5	(12.3-14.9)	3,647	19.6	(18.3-20.9)
**Western/Central Asia**	**HPV-16**	237	30.8	(25.0-37.1)	79	68.4	(56.9-78.4)
	**HPV-18**	237	6.8	(3.9-10.7)	79	6.3	(2.1-14.2)
	**HPV-31**	229	4.4	(2.1-7.9)	63	23.8	(14.0-36.2)
	**HPV-33**	210	7.1	(4.1-11.8)	31	9.7	(2.0-25.8)
	**HPV-45**	189	2.1	(0.6-5.8)	28	7.1	(0.9-23.5)
	**HPV-52**	72	2.8	(0.3-9.7)	20	0.0	(0.0-16.8)
	**HPV-58**	189	6.4	(3.3-10.8)	28	10.7	(2.3-28.2)
**South/Central America**	**HPV-16**	2,692	25.1	(23.5-26.8)	1,896	52.9	(50.6-55.1)
	**HPV-18**	2,690	6.8	(5.9-7.9)	1,886	9.4	(8.2-10.9)
	**HPV-31**	2,504	5.2	(4.4-6.1)	1,837	10.6	(9.2-12.1)
	**HPV-33**	2,517	7.5	(6.5-8.6)	1,776	6.5	(5.3-7.7)
	**HPV-45**	2,166	4.5	(3.7-5.5)	1,669	4.8	(3.8-5.9)
	**HPV-52**	2,127	5.1	(4.2-6.1)	1,587	6.3	(5.2-7.6)
	**HPV-58**	2,220	6.9	(5.8-8.0)	1,589	9.5	(8.1-11.1)
**North America**	**HPV-16**	5,532	24.7	(23.6-25.6)	4,598	56.7	(55.3-58.2)
	**HPV-18**	5,532	9.5	(8.7-10.3)	4,598	9.6	(8.8-10.5)
	**HPV-31**	5,532	8.9	(8.1-9.6)	4,598	13.1	(12.1-14.1)
	**HPV-33**	5,317	4.2	(3.7-4.8)	4,598	7.6	(6.9-8.4)
	**HPV-45**	5,415	5.6	(5.0-6.2)	4,397	4.8	(4.2-5.5)
	**HPV-52**	4,839	10.7	(9.9-11.6)	4,205	10.3	(9.4-11.3)
	**HPV-58**	5,213	7.6	(6.9-8.3)	4,397	6.8	(6.1-7.6)
**Europe**	**HPV-16**	14,319	25.9	(25.2-26.6)	8,348	54.4	(53.3-55.5)
	**HPV-18**	14,256	9.1	(8.6-9.6)	8,214	7.7	(7.2-8.3)
	**HPV-31**	13,166	12.5	(12.0-13.1)	7,930	12.4	(11.6-13.1)
	**HPV-33**	13,186	6.6	(6.2-7.1)	7,844	9.4	(8.7-10.0)
	**HPV-45**	10,472	4.6	(4.2-5.1)	6,244	3.8	(3.3-4.3)
	**HPV-52**	9,577	8.3	(7.8-8.9)	6,067	7.2	(6.6-7.9)
	**HPV-58**	10,489	6.7	(6.2-7.2)	6,278	5.8	(5.2-6.4)
**Oceania**	**HPV-16**	385	24.7	(20.5-29.3)	924	53.9	(50.6-57.2)
	**HPV-18**	385	8.6	(6.0-11.8)	924	9.6	(7.8-11.7)
	**HPV-31**	385	13.3	(10.0-17.0)	924	12.3	(10.3-14.6)
	**HPV-33**	385	5.7	(3.6-8.5)	924	7.9	(6.2-9.8)
	**HPV-45**	385	6.5	(4.3-9.4)	924	4.2	(3.0-5.7)
	**HPV-52**	385	11.4	(8.4-15.0)	924	12.2	(10.2-14.5)
	**HPV-58**	385	9.1	(6.4-12.4)	924	5.8	(4.4-7.6)

Low-grade, includes ASCUS, LSIL and CIN1; High-grade, includes HSIL, CIN2 and CIN3.

“RC”: Relative Contribution; “95%CI”: 95% Confidence Interval.

Additional information: HPV type specific relative contribution: Numerator = includes either single or multiple infections, thus the total sum of % can exceed 100%; Denominator = ”Tested” cases that stands for the HPV/DNA positive cases from studies testing for the HPV type in question, thus denominators vary by type.

The data source does not give specific information for HPV 6 and 11.

17. Data from Guan et al., IJC, 2012.

In the review of published papers in order to assess the RC of the 7 HR HPV types altogether for CIN2/3 or HSIL lesions, extraction of detailed data of single and multiple infections was possible in 4,101 cases (13 studies). Similar to ICC lesions, multiple infections were added to single types in accordance with a proportional weighting attribution. Multiple infections were higher in included studies compared to that in not included studies (41.3% vs 28.6%; p < 0,0001). Despite the consistency on the overall RC of the 9 HPV types observed in ICC, large variations between articles were observed for CIN2/3 or HSIL lesions, with RCs of the 7 HR HPV types ranging from 58.8% to 93.6%. Individual information on LR HPV 6/11 was available in 10 studies. Addition of HPV 6/11 did not modify the CIN2/3 or HSIL burden estimates (less than 1%).

## Discussion

We provided information on HPV distribution of 9 types included in a broad spectrum vaccine currently under development for 10 regions of the world and across different grades of cervical lesions. Although existing vaccines cover HPV 16/18 that are responsible of 70% of ICC worldwide, the inclusion of HR HPV 31/33/45/52/58 would increase the protection to almost 90% of the infections responsible for ICC.

As previously reported, HPV 16 is uniformly the most common HPV type in all histological types of cervical cancer and all the regions of the world [7,17]. Our findings confirmed the regional variations in the RCs of ICC due to each specific HPV type, with the most important differences observed in Eastern Asia, where HPV 52/58 are identified in more than 10% of cases, and the higher frequency of HPV 33 in Europe (6%), HPV 31 in South America (7%) and HPV 45 in Africa (10%) [7,17,22,23].

RC estimations in Africa from ICC study reflect that 52% of ICC cases were infected with an HPV type different from HPV 16. The different RCs on HPV genotypes in Africa are not fully understood. Different RCs may be related to host genetic determinants or could be the result of a different distribution on HPV types in HIV-infected women compared to that of the general population [24,25]. In Africa it should also be remarked the importance of HPV 35, identified in 5% of ICC cases in this region and ranking as the fourth most frequent type after HPV 16/18/45.

Our data confirm that HPV 16 is the most common type in all cervical lesions. By contrast, HPV 18/45 are underrepresented in HPV-positive women with precancerous cervical lesions compared to the high contribution in ICC, where these types are consistently the second and third most common types in most of the world regions [6,7,17]. Recent published data in high grade cervical lesions are in agreement with the reported results from Guan et al. meta-analysis, and suggests the representativeness of the study data [26].

It should be remarked that HPV 16/18/45 were detected at younger ages in ICC compared to other HPV types. Particularly for HPV 18/45, the detection at younger ages together with the underrepresentation in precancerous cervical lesions suggest a rapid progression to ICC of both HPV types, with or without transition through preinvasive stages [27,28].

Due to the restricted genotype contribution in the pathogenesis of ADC subtype, higher rates of protection with the vaccine could be expected, compared to SCC (95.5% vs 89.1%, respectively). Similar to previous findings a greater contribution of HPV 18/45 in ADC, ADSCC, and other histologies (such as neuroendocrine tumours) was observed compared to that in SCC and conversely, HPV 16 RC was higher in SCC [6,29-31]. Our data showed that HPV 16/18/45 were responsible for more than 94% of ADC. Much higher contributions of HPV 31/33/52/58 were observed in SCC (RC: 13.6%) and ADSCC (RC: 7.2%) compared to the RC of less than 1% observed in ADC cases. Only 4.5% of ADC cases were due to HPV types not included in the nine-valent vaccine. Therefore, this broad spectrum vaccine could help to reduce the increasing rates of cervical ADC reported in the last decades in some countries with established screening programmes, which is in keeping with the relative inability of cytological screening to reduce the rates of invasive ADC [32-34].

The existence of multiple infections complicates the estimation of the attribution of cases to HPV types. Assessment of RC of multiple infections was possible for ICC lesions, where multiple infections were added to single types in accordance with a proportional weighting attribution. We also calculated a more conservative RC by combining single type infections and multiple type infections restricted to the exact combination of the 9 mentioned types obtaining a RC close to 86% worldwide, meaning that the HPV types included in the nine-valent vaccine could cover the majority of infections responsible of ICC.

No similar attribution of multiple infections could be performed for precancerous cervical lesions from Guan et al. meta-analysis, meaning that HPV positive women with multiple infections could be counted more than once. Hence RCs for precancerous lesions was shown only for individual, and not combinations of HPV types. Although this approach does not allow to estimate type specific risk, it can be useful to identify HPV variations by region or lesion severity, as it has been confirmed that HPV type is the strongest factor that affects the risks of progression of precancerous cervical lesions given viral persistence, with HPV 16 and 18 less likely to regress than other HPV types [34].

CIN2/3 lesions rarely occur in absence of HR HPV infections [26,34,35], but this has not been confirmed in lower grades of cervical lesions. Data from Guan et al. meta-analysis reveal that only 52% of ASCUS cases were positive for HPV DNA compared to the more than 85% of positivity in CIN2/3 or HSIL lesions. We tried to estimate the combined RC of the 7 HR HPV types altogether for CIN2/3 or HSIL lesions similar to that in ICC obtaining a wide range from 58.8% to 93.6%., but due to the limitations of study designs, data should be interpreted cautiously and further research is needed to provide solid estimations taking into account attribution of multiple infections.

For estimations of HPV attributable cases, we assumed that current incidence rates will apply in the future, and calculated expected numbers using the appropriate population forecast. We did not take into account the impact of screening programs and current vaccines that will influence future rates since this type of analysis is more suitable for more complex analysis such as simulation models. Particularly for HPV vaccines it has to be taken into consideration that vaccination programs are not implemented worldwide, the coverage is still very low (especially in low income countries) and the impact on the reduction of invasive lesions will need more than ten years to be observable. The overall RC was used as the world AF. Even weighting regional estimations by world populations, the world attributable number of cases did not differ. Irrespective of changing risk, demographic changes will have a major impact in the future burden of ICC and this will be especially remarkable in developing countries where, in general incidence and mortality rates have been relatively stable over decades, reflecting the lack of screening implementation [32]. These estimates can help decision-makers evaluate the potential impact of existing and future vaccines worldwide providing an estimation of the proportion of HPV related cancer that could be avoided if the populations become immune to the 9 types of HPV included in the vaccine.

The good concordance of HPV estimations with previous reports suggests the representativeness of the study data, and especially in those regions with large sample sizes. Eighty five percent of all ICC from the ICC study were positive for DNA, compared to the ninety percent estimated in the most recent IARC meta-analysis on ICC available in the literature [6]. Given that HPV is accepted as a necessary cause of ICC, the negativity of both studies is expected to be related to technical reasons, but also to the quality of the biological specimen.

The low number of cases in the ICC study from Oceania and North America could be a potential limitation. Although RCs for Oceania are similar to that observed in previous reports, estimations of RCs for North America based on this study differ to that observed in the IARC meta-analysis and the largest study performed in this region by Wheeler et al. [6,36], with higher rates for HPV 16 (72% vs 55% and 53% respectively) and lower for HPV 18 (7% vs 18% and 13% respectively). RC for the other HPV types included in the vaccine was similar between studies. A possible explanation could be the different year of diagnosis of the cases. Wheeler et al. observed that the proportion of HPV 16 positive cases declined with more recent calendar year of diagnosis. On the other hand, estimates from the meta-analysis and Wheeler et al. study are based on all cases and not on HPV DNA positive cases. In order to assess these differences we recalculated estimations for North America using all tested women as a denominator and although RC of HPV 16 decreased, it was still higher in the included ICC study.

Irrespective of histological type and similar to previous reports [37], HPV 16/18/45 were identified in younger women more than other HPV types. Combined with the low proportion of ICC cases attributable to HPV types not included in the nine-valent broad spectrum vaccine, it suggests that such intervention would imply a drastic reduction of screening intervention in vaccinated cohorts.

## Conclusions

The inclusion of additional HPV types in nine-valent vaccine currently under development could have direct implications for cervical cancer incidence and prevention in all regions of the world. If the nine-valent vaccine achieves the same degree of efficacy as has been shown for HPV 16 and 18, and vaccination programs are effectively implemented, almost the 90% of ICC cases worldwide could be prevented. This means that Globocan 2008 world incidence rates could be substantially reduced. If all our estimations are true, and assuming high vaccination coverage and no HPV type replacement, this intervention would provide a higher impact than any screening programme has ever produced in the very best scenarios.

## Competing interests

**GMC** has no potential conflict of interest. **BS** and **ST:** Institutional support: HPV vaccine trials and epidemiological studies sponsored by GlaxoSmithKline, Merck and Sanofi Pasteur MSD. **LA:** Institutional support: HPV vaccine trials and epidemiological studies sponsored by GlaxoSmithKline, Merck and Sanofi Pasteur MSD. Personal support: Travel grants to conferences occasionally granted by Merck and Sanofi Pasteur MSD. **LB:** Institutional support: HPV vaccine trials and epidemiological studies sponsored by GlaxoSmithKline, Merck and Sanofi Pasteur MSD. Travel grants to conferences occasionally granted by Sanofi Pasteur MSD. **TW:** Is currently working at Merck. **FXB:** Institutional support: HPV vaccine trials and epidemiological studies sponsored by GlaxoSmithKline, Merck and Sanofi Pasteur MSD. Personal support: Travel grants to conferences / symposia / meetings and honorarium are occasionally granted by either GlaxoSmithKline, Merck, Sanofi Pasteur MSD, Roche or Qiagen. **SS:** Institutional support: HPV vaccine trials and epidemiological studies sponsored by GlaxoSmithKline, Merck and Sanofi Pasteur MSD. Personal support: Travel grants to conferences / symposia / meetings are occasionally granted by either GlaxoSmithKline, Sanofi Pasteur MSD or Qiagen.

## Authors' contributions

BS, LA and ST were responsible for the data analysis. All authors contributed to the writing of the manuscript and BS was responsible for the preparation of the manuscript for submission. All authors read and approved the final manuscript.
